# Causal Association Between Periodontitis and Type 2 Diabetes: A Bidirectional Two-Sample Mendelian Randomization Analysis

**DOI:** 10.3389/fgene.2021.792396

**Published:** 2022-01-10

**Authors:** Yong-Bo Wang, Si-Yu Yan, Xu-Hui Li, Qiao Huang, Li-Sha Luo, Yun-Yun Wang, Jiao Huang, Ying-Hui Jin, Xian-Tao Zeng

**Affiliations:** ^1^ Department of Stomatology, Zhongnan Hospital of Wuhan University, Wuhan, China; ^2^ Center for Evidence-Based and Translational Medicine, Zhongnan Hospital of Wuhan University, Wuhan, China; ^3^ Department of Geriatrics, Zhongnan Hospital of Wuhan University, Wuhan, China

**Keywords:** periodontitis, oral health, type 2 diabetes, Mendelian randomization, single-nucleotide polymorphisms

## Abstract

**Background:** Previous observational studies have reported a bidirectional association between periodontitis and type 2 diabetes, but the causality of these relationships remains unestablished. We clarified the bidirectional causal association through two-sample Mendelian randomization (MR).

**Methods:** We obtained summary-level data for periodontitis and type 2 diabetes from several published large-scale genome-wide association studies (GWAS) of individuals of European ancestry. For the casual effect of periodontitis on type 2 diabetes, we used five independent single-nucleotide polymorphisms (SNPs) specific to periodontitis from three GWAS. The summary statistics for the associations of exposure-related SNPs with type 2 diabetes were drawn from the GWAS in the Diabetes Genetics Replication and Meta-analysis (DIAGRAM) consortium and the FinnGen consortium R5 release, respectively. For the reversed causal inference, 132 and 49 SNPs associated with type 2 diabetes from the DIAGRAM consortium and the FinnGen consortium R5 release were included, and the summary-level statistics were obtained from the Gene-Lifestyle Interactions in Dental Endpoints consortium. Multiple approaches of MR were carried out.

**Results:** Periodontitis was not causally related with the risk of type 2 diabetes (all *p* > 0.05). No causal effect of type 2 diabetes on periodontitis was found (all *p* > 0.05). Estimates were consistent across multiple MR analyses.

**Conclusion:** This study based on genetic data does not support a bidirectional causal association between periodontitis and type 2 diabetes.

## 1 Introduction

Periodontitis is an inflammatory disease of the gums with worldwide influence (Tonetti et al., 2017; Fang et al., 2021b). The prevalence rate of periodontitis is estimated to be high, with around 20–50% in the general population (Dye, 2012; Luo et al., 2021). Type 2 diabetes mellitus is the most common form of diabetes mellitus, comprising 90% of cases, and is considered a metabolic disorder (Zheng et al., 2018). There is an indication that patients with type 2 diabetes are more likely to suffer from dental diseases (such as periodontitis) (Bharateesh et al., 2012). In addition, studies have promoted that periodontitis is a risk factor for type 2 diabetes (Ide et al., 2011). Both of these two diseases may be driven by inflammatory processes, which may be a potential explanation for this bidirectional relationship (Ide et al., 2011). The relationship between these two diseases is complex because type 2 diabetes is a metabolic disorder associated with a cluster of conditions collectively known as metabolic syndrome. Meanwhile, an increasing body of evidence suggests that periodontitis may also belong to the metabolic syndrome cluster (Winning and Linden, 2017). Meta-analysis summarized the findings of glucose disorders (including periodontitis and type 2 diabetes) and showed a positive correlation between these two factors (Nascimento et al., 2018). There have been several observational studies reporting a possible bidirectional association between periodontitis and type 2 diabetes (Taylor, 2001; Winning and Linden, 2017; Genco et al., 2020; Stöhr et al., 2021). Because of the restriction of methodological bias, such as residual confounding, reverse causation, and measurement error, it is difficult to determine in conventional observational studies whether these correlations are causal. Besides, randomized controlled trials (RCTs) are ethically impermissible and it is impractical to observe the causality of these relationships. To draw a clear conclusion on these associations, effective methods that consider methodological challenges are needed.

Mendelian randomization (MR) analysis can be trusted using summary data from genome-wide association studies (GWAS) to assess the causality in the putative exposure–outcome pathway (Burgess et al., 2015). Because the genetic variation is randomly arranged in meiosis and fixed after fertilization, this technology can reduce the residual confounding factors and reverse causality. In this way, grouped by the naturally allocated genetic instrumental variables (IVs), the MR simulates an RCT employing an individual or summary-level data from observational studies. Two-sample MR analysis is a widely used method of MR that allows MR analyses to be performed using GWAS summary statistics, rather than limiting them to using personal-level data from a sample. We conducted a bidirectional two-sample MR study to explore the causality and causal direction between periodontitis and type 2 diabetes, using publicly available summary statistics from GWAS.

## 2 Materials and Methods

### 2.1 Study Design

**FIGURE 1:**
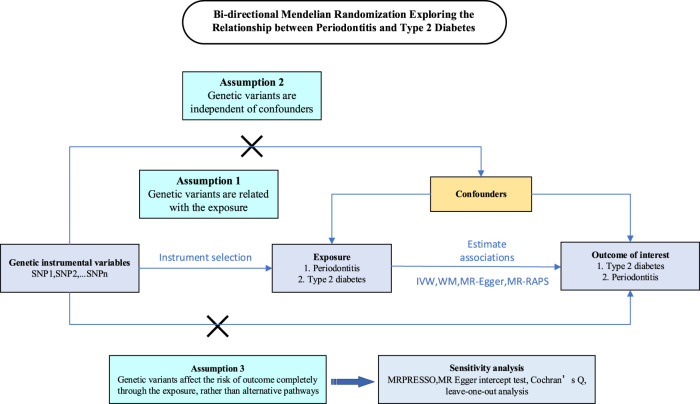
Schematics for the bidirectional Mendelian randomization design. Mendelian randomization requires valid genetic instrumental variants satisfying three assumptions.

Our bidirectional two-sample MR study was conducted in a framework shown in Figure 1. Genetic variants were employed to explore the causal effect of periodontitis and type 2 diabetes and the reverse causation separately. In order to gain reliable results, the effective IVs must satisfy three key assumptions during the MR analysis process (Davey Smith and Hemani, 2014; Davey Smith et al., 2020): 1) the IVs are strongly associated with the exposure, 2) the IVs are not related to any confounding factors that affect both exposure and outcome, and 3) the IVs influence outcome only through the exposure. For each inference direction, the analysis includes three main procedures: selecting the appropriate IVs for the exposure of interest, applying multiple MR methods, and performing pleiotropic effect analyses.

### 2.2 Data Sources

#### 2.2.1 Genetic Instrument Selection

Our MR study was performed using publicly published studies or shared datasets. No additional ethics statement or consent was required. For the first assumption, only SNPs associated with exposure interest at the genome-wide significance threshold (*p* < 5 × 10^–8^) from a GWAS or a meta-analysis of GWAS were included. In addition, linkage disequilibrium (LD) among SNPs for one exposure of interest was evaluated according to the 1,000 genomes data from the European individuals and defined by *r* ^2^ > 0.001 or clump distance <10 kb. When multiple SNPs were confirmed at the same locus, only the “leader” SNP (i.e., with the smallest *p*-value) was included.

In addition, the assumptions of IVs being independent of confounding factors and outcome were investigated for the genome-wide significant associations (*p* < 5 × 10^–8^) with confounding factors and their corresponding outcome by searching the PhenoScanner V2 website (http://www.phenoscanner.medschl.cam.ac.uk/). We searched for potential confounding factors which might affect periodontitis and type 2 diabetes risk simultaneously (Chapple et al., 2017). Finally, six potential confounding factors were identified, including smoking, alcohol, body mass index, obesity, rheumatoid arthritis, and micronutrient deficiencies. Therefore, every SNP was searched in PhenoScanner V2 to check for signs of pleiotropy, and the SNPs associated with potential confounders or outcome variables at genome-wide significance (*p* < 5 × 10–8) were removed to satisfy these two assumptions. The search results were limited to Europeans only.

#### 2.2.2 MR of Periodontitis on Risk of Type 2 Diabetes

Only SNPs correlated with periodontitis met the genome-wide significance level (*p* < 5 × 10^–8^) in a meta-analysis of GWAS or GWAS were included. Studies that reported only rare variants were excluded, as were studies that relied on self-reported phenotypes. We focused on populations of European descent to match the population demographics of our outcome data. Finally, the IVs for periodontitis data were identified from three recent GWAS of clinically confirmed periodontitis in Dutch, German, or European-American samples (Schaefer et al., 2010; Munz et al., 2017; Munz et al., 2019). Candidate SNPs were assessed for suitability against the assumptions required of a valid IV described before.

The Diabetes Genetics Replication and Meta-analysis (DIAGRAM) consortium provided the summary-level data for type 2 diabetes. The consortium included 32 studies with a total of 898,130 participants of European descent (74,124 cases and 824,006 controls) (Mahajan et al., 2018). The average age of the individuals was about 55 y, and 51.8% were men. Data from the FinnGen consortium R5 release (32,469 cases and 183,185 controls) were used in the replication phase. Detailed methods (such as data collection, participating cohorts, genotyping, and data analysis) are provided on its webpage (https://www.finngen.fi/fi). Sex, age, and genetic principal components were adjusted in the association tests in both sources.

#### 2.2.3 MR of Type 2 Diabetes on Risk of Periodontitis

The IVs for evaluating the causal effect of type 2 diabetes on the risk of periodontitis were derived from the DIAGRAM consortium and the FinnGen consortium R5 release with respective summary statistics (Supplementary Tables S3,4). For our study, summary-level data for periodontitis were available from the Gene-Lifestyle Interactions in Dental Endpoints (GLIDE) consortium by Shungin et al. (Shungin et al., 2019). The data of people with European ancestry were only used and excluded those with Hispanic/Latino backgrounds in the GLIDE consortium (12,289 clinically diagnosed cases and 22,326 controls).

### 2.3 Statistical Analyses for MR Estimates

Two-sample MR was performed for chosen SNPs’ individual lookup requests against multiple target GWAS, harmonization of effect allele across studies, LD pruning, and sensitivity analyses. Where possible, SNPs that were not present in the outcome data were replaced by proxy SNPs in the high LD from the 1,000 Genome Project European data. The proxies needed to have a minimum *R* ^2^ value of 0.8 (Rasooly and Patel, 2019). Odds ratios (ORs) and confidence intervals (CIs) were scaled to one-unit increment of ln OR of periodontitis and type 2 diabetes. We used an F-statistic to estimate the strength of each chosen SNP (Palmer et al., 2012) and calculate the variance explained by each IV in the exposure. (The method for F-statistic computations is shown in Supplementary Table S1; F-statistic < 10 was deemed as a weak instrument.)

When the effect of the variant on the outcome exceeds its effect on exposure in MR, horizontal pleiotropy would occur. To test for the existence of potential pleiotropic effects, we used the MR–pleiotropy residual sum and outlier (MR-PRESSO) test to identify outlier SNPs (Verbanck et al., 2018). In MR-PRESSO, the inverse variance weighting (IVW) method is implemented by regression, and the residual sum of squares is calculated as a measure of heterogeneity. If the residual sum of squares is reduced compared to the simulated expected distribution, the SNP is removed from the analysis. The causal relationship in both conditions was tested by the following MR effect estimation methods: the IVW method (fixed and random effects) (Burgess et al., 2013), weighted median (Bowden et al., 2016), and MR-Egger (Bowden et al., 2015). The IVW method uses beta coefficients and standard errors combined with risk factors and regresses the results of every genetic variation, in turn, using summarized data from all the genetic variations to assess causality (Burgess et al., 2013). The weighted median method can perform consistency analysis by combining data from multiple genetic instruments by calculating a single weighted median estimator (Bowden et al., 2016). The MR-Egger method allows each IV to exhibit pleiotropy, and if the instrument strength is not related to these pleiotropic effects, the method is consistent (Bowden et al., 2015). In addition, we also evaluated the causal effect via the MR-RAPS (robust adjusted profile score) method, due to its robustness to weak instrument deviations (Zhao et al., 2018). The MR-Egger regression can inspect the horizontal pleiotropy through its intercept and provides estimates after correcting for the pleiotropic effects, although it consumes statistical power (Bowden et al., 2015). Cochran’s Q verified the heterogeneity between the causal estimates of each SNPs in the IVW and MR-Egger methods. To identify whether the effect estimate was caused by a particular single SNP, we conducted leave-one-out analysis with each SNP removed.

Statistical analysis was performed using R Software (version 3.6.1), through MR-PRESSO (1.0) (Verbanck et al., 2018) and TwoSampleMR (0.5.5) (Hemani et al., 2018) packages, and *p* < 0.05 was considered statistically significant.

### 2.4 Sample Size and Power Calculations

Power calculations were performed in *mRnd*, a Web-based application (https://shiny.cnsgenomics.com/mRnd/) (Brion et al., 2013) assuming a 5% type I error rate. Statistical significance was set at a two-sided *α* of 0.05. Power calculations are given in Supplementary Table S6.

## 3 Results

After the selection of SNPs with *p* < 5 × 10^–8^, pairwise LD clumping, matching of coding alleles between the summary statistics of the exposure and those of the outcome, and removal of SNPs associated with potential confounders, the valid IVs were chosen to fit the previous three basic MR assumptions. A total of five SNPs strongly associated with periodontitis were selected as IVs (Supplementary Tables S1,2). In reversed MR analysis, 165 and 62 SNPs significantly associated with type 2 diabetes, respectively, were included (Supplementary Tables S3,4).

Among the five periodontitis-associated SNPs, all were available in the two datasets of type 2 diabetes (DIAGRAM and FinnGen consortiums). Using the five independent SNPs for periodontitis, the average F-statistic was 4.89, which suggests relatively weak instruments in this study. The statistical power to detect an OR of 1.05 was 99 and 37% in the analysis of type 2 diabetes from GWAS in the DIAGRAM and FinnGen consortiums, respectively (Supplementary Table S6).

**FIGURE 2:**
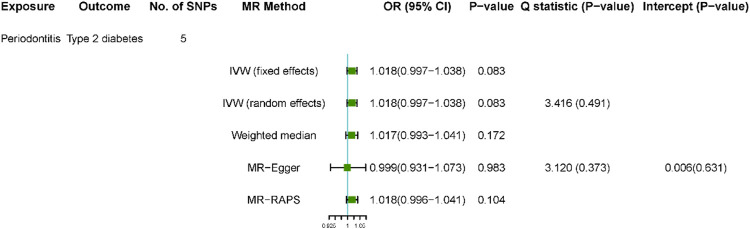
Estimated causal effect of periodontitis on type 2 diabetes using different MR approaches. Using five instrumental SNPs for periodontitis and the summary statistics were obtained from DIAGRAM.

**FIGURE 3:**
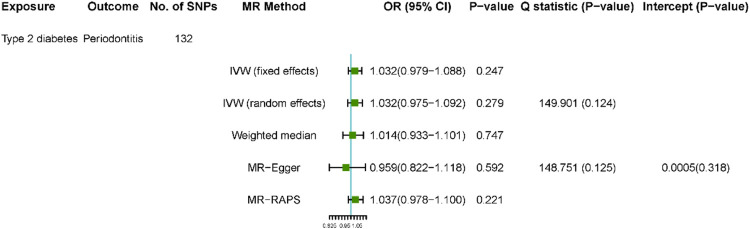
Estimated causal effect of type 2 diabetes on periodontitis. Using 132 instrumental SNPs from DIAGRAM for type 2 diabetes.

The results of the bidirectional MR estimates are presented in Figures 2, 3. Genetic liability to periodontitis was not significantly associated with type 2 diabetes risk. The OR of type 2 diabetes per one-unit increase in lnOR of periodontitis was 1.018 (95% confidence interval [CI] 0.997–1.038) and 1.018 (95%CI 0.997–1.038) in the DIAGRAM consortium based on the IVW fixed method, which was consistent in the weighted median method (OR 1.017, 95% CI 0.993–1.041) (Figure 2). In addition, the MR estimate using the MR-RAPS method was similar (OR 1.018, 95% CI 0.996–1.041). The MR-Egger estimate was less consistent though (OR 0.999, 95% CI 0.931–1.073). The validation using FinnGen as outcome confirmed the aforementioned results (Supplementary Figure S1).

After SNP exclusion and proxy replacement, we, respectively, used 132 and 49 SNPs for type 2 diabetes as instrumental variables in the analyses of the effects of type 2 diabetes on periodontitis in the DIAGRAM and FinnGen consortiums. Using 132 or 49 SNPs as instruments for type 2 diabetes, the average F-statistics were 26.74 vs. 22.58, respectively, which indicated considerable weak instrument bias would not be expected (Supplementary Tables S3,4). We had high power (100% power to detect an OR of 1.1) to detect weak associations of type 2 diabetes with periodontitis (Supplementary Table S6). Using the 132 SNPs as instruments for type 2 diabetes, there was no relationship between type 2 diabetes and the risk of periodontitis (OR 1.032, 95% CI 0.979 to 1.088; OR 1.032, 0.975–1.092) from the IVW fixed and random methods (Figure 3). The relationships of periodontitis with type 2 diabetes biomarkers were consistent in other sensitivity analyses. In addition, the validation using 49 SNPs as instruments from the FinnGen consortium confirmed the aforementioned results (Supplementary Figure S2).

No outliers in the bidirectional MR analyses were observed with MR-PRESSO (global test *p* > 0.11 for all) (Supplementary Table S5). No substantial evidence for horizontal pleiotropy was detected in the MR-Egger regression analyses in all analyses (Figures 2, 3). This was also indicated in the scatterplots (Supplementary Figures S3–6). There was no evidence for heterogeneity between SNPs evaluated by Cochran’s Q statistic. The results of the leave-one-out analyses did not indicate that the effects were disproportionately influenced by a single SNP (Supplementary Figures S7–10).

## 4 Discussion

We explored the potential causal association of periodontitis in the risk of type 2 diabetes and the reverse causal relationship of type 2 diabetes with the development of periodontitis using multiple MR approaches. As a whole, the results do not support a bidirectional association between these two diseases. Nevertheless, given the paucity of GWAS on these two diseases to date, these results should be interpreted with caution.

There are several possible explanations for the bidirectional associations between periodontitis and type 2 diabetes in observational studies. One possible explanation for this association is that diabetes may directly influence the oral microbiome, resulting in a state of dysbiosis (Polak et al., 2020). However, the most studied explanation involves the inflammatory pathway (Mao et al., 2013; Genco et al., 2020; Genco and Sanz, 2020; Fang et al., 2021a; Yuan et al., 2021). Inflammatory markers have been shown repeatedly to be elevated in the presence of these two comorbidities (Noack et al., 2001; Genco et al., 2005; Polak et al., 2020). Studies have suggested that periodontal therapy can have a positive effect on glycated hemoglobin levels in the blood by reducing the periodontal inflammatory load. On the other hand, it has been shown that Gram-negative bacteria in the periodontal pocket can increase serum inflammatory markers, such as c-reactive protein (Noack et al., 2001). This could highly attract inflammatory immune cells and boost the release of pro-inflammatory cytokines, which result in insulin resistance (Genco et al., 2005).

A meta-analysis including 15 cohort studies observed that the risk of periodontitis increased by 24% for patients with diabetes, and for patients with periodontitis, the relative risk of developing diabetes mellitus was increased by 26% (Stöhr et al., 2021). Nascimento et al.’s meta-analysis (Nascimento et al., 2018) found an 86% elevated relative risk of periodontitis for populations with diabetes. Another meta-analysis by Ziukaite et al. (Ziukaite et al., 2018) concentrated on the other direction of the relation and detected a 27% higher prevalence of diabetes for individuals with periodontitis.

The risk of bias in these meta-analyses was high due to different definitions of exposure and outcomes and different study designs of included studies. In cohort studies, although the onset of exposure can be detected before or after the outcome, it is difficult to assess causality because of the affection of reverse causality or confounding effects. In addition, confounding may provide an explanation as to why a causal relationship was not found in our study, despite observational studies suggesting otherwise. Periodontitis and type 2 diabetes are both multifactorial diseases that manifest in individuals who have been exposed to risk factors for many years. Therefore, the observed relationship between these two diseases may be confused by a range of other effects (Haworth et al., 2021). For example, obesity is a vital risk factor shared by both periodontitis and type 2 diabetes (Lavigne and Forrest, 2021). Other possible confounders include smoking, alcohol, rheumatoid arthritis, and micronutrient deficiencies. However, our data do not support a bidirectional causal association between periodontitis and type 2 diabetes based on strict genetic instrument selections and multiple MR analyses.

The current study had several strengths. First, MR design reduces the residual confounding and other biases, thus strengthening the causal inference. This bidirectional MR analysis ensures the inference of causality between periodontitis and type 2 diabetes in both directions. We evaluated the relationships in two independent populations, and the high degree of consistency made our research steady. Second, we conducted several sensitivity analyses. The consistent estimation of different models strengthened our confidence in the established associations. Third, we applied the latest GWAS of periodontitis and type 2 diabetes in the population of European descent to obtain sufficient statistical power to assess the potential causal relationship between periodontitis and type 2 diabetes, thus minimizing the impact of population stratification. The greater advantage was the large sample size used for these two results (so there is enough statistical power to detect even relatively weak causal effects).

However, there are still a number of limitations that need to be discussed. First, MR uses genetic variation to affect the average risk of a particular characteristic during the participant’s lifetime; in this case, it cannot answer whether exposure during a particular life cycle has any effect on the risk of the outcome. Second, the deficiency of GWAS in other populations limits the generality of our results. Third, at the genome-wide significance level, only five independent SNPs related with clinically defined periodontitis were determined to be included in this study. Considering the number of GWAS that have been performed on this disease, this is a bit small. However, exploring the small effects between these feature pairs based on weak tool deviations may be limited. The weak instruments have a tendency to shift the MR estimate toward the null in two-sample MR (Davies et al., 2018), which may lead to uncertain causality between periodontitis and type 2 diabetes in our study. Fourth, these five SNPs were all detected in cohorts that only included patients with aggressive periodontitis or patients with a mix of both chronic and aggressive periodontitis. Although chronic periodontitis is the main form of this disease, GWAS only using the chronic periodontitis cohort failed to identify any significant genome-wide variation related with the disease (Divaris et al., 2013; Teumer et al., 2013; Feng et al., 2014; Shaffer et al., 2014; Hong et al., 2015; Shimizu et al., 2015). Importantly, because the definition of periodontitis used in different studies is inconsistent, GWAS on periodontitis tend to fail in identifying consistent SNPs. More importantly, these three GWAS were different in gene chip, quality control, and data analysis, which may cause heterogeneity, whereas we did not find significant heterogeneity among five SNPs by Cochran’s Q statistic. And the results of the leave-one-out analyses did not indicate that the effects were disproportionately influenced by a single SNP. Fifth, one important limitation is pleiotropy, whereas we did not find any possible pleiotropic effects in the MR-Egger regression and outliers in the MR-PRESSO analysis. Sixth, we emphasize that the calculation of MR estimates associated with binary exposures (unlike continuous exposures) is more effective in determining the presence of a causal effect than in quantifying the size of the causal effect (Burgess and Labrecque, 2018). We emphasize that biological mechanisms of how these SNPs affect periodontitis and type 2 diabetes are still imprecise.

In conclusion, in this bidirectional MR study, we did not find robust evidence to support a bidirectional causal effect between periodontitis and type 2 diabetes from the GWAS results within large-scale populations with European ancestry. This result is in contrast to observational studies, which have shown an association between these diseases. Furthermore, GWAS research is needed to explore the consistency of these results.

## Data Availability

The original contributions presented in the study are included in the article/Supplementary Material; further inquiries can be directed to the corresponding authors.
