# Supplementary material for: Causal Association Between Periodontitis and Type 2 Diabetes: A Bidirectional Two-Sample Mendelian Randomization Analysis
Source: Front Genet. 2022 Jan 10;12:792396. doi: 10.3389/fgene.2021.792396 (PMC8784400; doi:10.3389/fgene.2021.792396)
Supplement: Supplementary file 1 [file DataSheet1.docx]

Supplementary Tables and Figures

**Supplementary Table 1.** Genetic instrumental variables utilized in the Mendelian randomization analysis of periodontitis on type 2 diabetes (the GWAS of type 2 diabetes were obtained from DIAGRAM)

**Supplementary Table 2.** Genetic instrumental variables utilized in the Mendelian randomization analysis of periodontitis on type 2 diabetes (the GWAS of type 2 diabetes were obtained from FinnGen)

**Supplementary Table 3.** Summary statistics for Mendelian randomization analysis of potential causal effect of type 2 diabetes (DIAGRAM) on periodontitis (GLIDE)

**Supplementary Table 4.** Summary statistics for Mendelian randomization analysis of potential causal effect of type 2 diabetes (FinnGen) on periodontitis (GLADE)

**Supplementary Table 5.** MR-PRESSO global test

**Supplementary Table 6.** Post-hoc power calculations for Mendelian randomization analyses performed at varying causal effect sizes

**Supplementary Figure 1.** Estimated causal effect of periodontitis on type 2 diabetes using different MR approaches.

**Supplementary Figure 2.** Estimated causal effect of type 2 diabetes on periodontitis. Using 49 instrumental SNPs from FinnGen for type 2 diabetes

**Supplementary Figure 3.** Scatterplots comparing the strength of the SNP-exposure (periodontitis) and SNP-type 2 diabetes (DIAGRAM) associations

**Supplementary Figure 4.** Scatterplots comparing the strength of the SNP-exposure (periodontitis) and SNP-type 2 diabetes (FinnGen) associations

**Supplementary Figure 5.** Scatterplots comparing the strength of the SNP-exposure (type 2 diabetes in DIAGRAM) and SNP-periodontitis associations

**Supplementary Figure 6.** Scatterplots comparing the strength of the SNP-exposure (type 2 diabetes in FinnGen) and SNP-periodontitis associations

**Supplementary Figure 7-10**. Leave-one-out meta-analysis

**Supplementary Table 1.** Genetic instrumental variables utilized in the Mendelian randomization analysis of periodontitis on type 2 diabetes (the GWAS of type 2 diabetes were obtained from DIAGRAM)

| SNP | Chr | Nearest genes/contig | Effect allele | Other allele | Eaf | Exposure: periodontitis | | | Outcome: type 2 diabetes^*^  (74,124 cases and 824,006 controls) | | | R^2^ | F | Potential confounders |
| --- | --- | --- | --- | --- | --- | --- | --- | --- | --- | --- | --- | --- | --- | --- |
|  |  |  |  |  |  | Coefficient^#^ | SE | *P* value | Coefficient^#^ | SE | *P* value |  |  |  |
| Schaefer et al. [1] (periodontitis 142 cases vs. 472 controls) | | | | | | | | | | | |  |  |  |
| rs1537415 | 9 | *GLT6D1* | C | G | 0.41 | 0.464 | 0.080 | 5.51E-09 | 0.0037 | 0.0065 | 0.57 | 0.022 | 6.713 |  |
| Munz *et al.* [2] (periodontitis 4,924 cases vs. 7,301 controls) | | | | | | | | | | | |  |  |  |
| rs16870060 | 8 | MTND1P5 | G | T | 0.91 | 0.307 | 0.0521 | 3.69E-09 | 0.0170 | 0.0110 | 0.13 | 0.015 | 2.914 |  |
| rs729876 | 16 | LOC107984137 | T | C | 0.90 | 0.218 | 0.0380 | 9.77E-09 | -0.0042 | 0.0081 | 0.60 | 0.009 | 0.895 |  |
| Munz et al.[3] (periodontitis 851 cases vs. 6,580 controls) | | | | | | | | | | | |  |  |  |
| rs4284742 | 19 | SIGLEC5 | G | A | 0.76 | 0.293 | 0.051 | 1.34E-08 | 0.0081 | 0.0076 | 0.29 | 0.031 | 7.294 |  |
| rs2738058 | 8 | DEFA1A3 | T | C | 0.43 | 0.247 | 0.038 | 6.78E-10 | 0.0097 | 0.0064 | 0.13 | 0.030 | 6.650 |  |

Abbreviations: Chr: chromosome; Eaf: effect allele frequency; SE: standard error; SNP: single-nucleotide polymorphism; R^2^, proportion of variance explained.

^*^ the GWAS of type 2 diabetes were obtained from DIAGRAM; ^#^Coefficient: ln (odds ratio)

**References**

1. Schaefer, A.S., et al., *A genome-wide association study identifies GLT6D1 as a susceptibility locus for periodontitis.* Hum Mol Genet, 2010. **19**(3): p. 553-62.

2. Munz, M., et al., *Meta-analysis of genome-wide association studies of aggressive and periodontitis identifies two novel risk loci.* Eur J Hum Genet, 2019. **27**(1): p. 102-113.

3. Munz, M., et al., *A genome-wide association study identifies nucleotide variants at SIGLEC5 and DEFA1A3 as risk loci for periodontitis.* Hum Mol Genet, 2017. **26**(13): p. 2577-2588.

**Supplementary Table 2.** Genetic instrumental variables utilized in the Mendelian randomization analysis of periodontitis on type 2 diabetes (the GWAS of type 2 diabetes were obtained from FinnGen)

| SNP | Chr | Nearest genes/contig | Effect allele | Other allele | Eaf | Exposure: periodontitis | | | Outcome: type 2 diabetes^†^  (32,469 cases and 183,185 controls) | | | Potential confounders |
| --- | --- | --- | --- | --- | --- | --- | --- | --- | --- | --- | --- | --- |
|  |  |  |  |  |  | Coefficient^#^ | SE | *P* value | Coefficient^#^ | SE | *P* value |  |
| Schaefer et al. [1] (periodontitis 142 cases vs. 472 controls) | | | | | | | | | | | |  |
| rs1537415 | 9 | *GLT6D1* | C | G | 0.41 | 0.464 | 0.080 | 5.51E-09 | 0.0077 | 0.0108 | 0.4738 |  |
| Munz *et al.* [2] (periodontitis 4,924 cases vs. 7,301 controls) | | | | | | | | | | | |  |
| rs16870060 | 8 | MTND1P5 | G | T | 0.91 | 0.307 | 0.0521 | 3.69E-09 | 0.0047 | 0.0153 | 0.7576 |  |
| rs729876 | 16 | LOC107984137 | T | C | 0.90 | 0.218 | 0.0380 | 9.77E-09 | 0.0081 | 0.0143 | 0.5694 |  |
| Munz et al.[3] (periodontitis 851 cases vs. 6,580 controls) | | | | | | | | | | | |  |
| rs4284742 | 19 | SIGLEC5 | G | A | 0.76 | 0.293 | 0.051 | 1.34E-08 | -0.0053 | 0.0117 | 0.6497 |  |
| rs2738058 | 8 | DEFA1A3 | T | C | 0.43 | 0.247 | 0.038 | 6.78E-10 | 0.0039 | 0.0104 | 0.7104 |  |

Chr: chromosome; Eaf: effect allele frequency; SE: standard error; SNP: single-nucleotide polymorphism, R^2^, proportion of variance explained.

^†^ the GWAS of type 2 diabetes were obtained from FinnGen; ^#^Coefficient: ln (odds ratio)

**References**

1. Schaefer, A.S., et al., *A genome-wide association study identifies GLT6D1 as a susceptibility locus for periodontitis.* Hum Mol Genet, 2010. **19**(3): p. 553-62.

2. Munz, M., et al., *Meta-analysis of genome-wide association studies of aggressive and periodontitis identifies two novel risk loci.* Eur J Hum Genet, 2019. **27**(1): p. 102-113.

3. Munz, M., et al., *A genome-wide association study identifies nucleotide variants at SIGLEC5 and DEFA1A3 as risk loci for periodontitis.* Hum Mol Genet, 2017. **26**(13): p. 2577-2588.

**Supplementary Table 3.** Summary statistics for Mendelian randomization analysis of potential causal effect of type 2 diabetes (DIAGRAM) on periodontitis (GLIDE)

| SNP | Chr | Nearest genes/contig | Effect allele | Other allele | Eaf† | Exposure: type 2 diabetes^*^ | | | Outcome: periodontitis^†^  (21,982 cases vs. 41,944 controls) | | | Potential confounders |
| --- | --- | --- | --- | --- | --- | --- | --- | --- | --- | --- | --- | --- |
|  |  |  |  |  |  | Coefficient^#^ | SE | P value | Coefficient^#^ | SE | P value |  |
| rs1005752 | 15 | RP11-307C19.1 | A | C | 0.715 | 0.077 | 0.007 | 3.00E-29 | -0.006 | 0.0168 | 0.7219 |  |
| rs10096633 | 8 | LPL | T | C | 0.123 | -0.068 | 0.009 | 1.00E-12 | -0.0121 | 0.0226 | 0.5928 |  |
| rs10097617 | 8 | TP53INP1 | T | C | 0.485 | 0.039 | 0.006 | 3.00E-11 | 0.0116 | 0.0154 | 0.4515 |  |
| rs10193538 | 2 | LINC01122 | T | G | 0.610 | 0.039 | 0.007 | 9.00E-09 | 0.0075 | 0.0156 | 0.6291 | BMI |
| rs10195252 | 2 | COBLL1 | T | C | 0.586 | 0.068 | 0.007 | 6.00E-25 | -0.0251 | 0.0229 | 0.2715 |  |
| rs10228066 | 7 | GTF3AP5 | T | C | 0.537 | 0.068 | 0.006 | 1.00E-28 | 0.0091 | 0.0153 | 0.5516 |  |
| rs10406327 | 19 | PEPD | C | G | 0.523 | 0.039 | 0.007 | 4.00E-08 | 0.0213 | 0.0156 | 0.1714 |  |
| rs10406431 | 19 | RN7SL836P | A | G | 0.563 | 0.049 | 0.007 | 1.00E-13 | 0.0223 | 0.0166 | 0.1807 |  |
| rs1061810 | 11 | HSD17B12 | A | C | 0.288 | 0.049 | 0.007 | 6.00E-13 | -0.0106 | 0.0168 | 0.5279 |  |
| rs10750397 | 11 | ETS1 | A | G | 0.282 | 0.049 | 0.007 | 8.00E-13 | -0.0196 | 0.0167 | 0.2421 |  |
| rs10811660 | NA | CDKN2B-AS1 | A | G | 0.172 | -0.239 | 0.010 | 1.00E-115 | -0.0153 | 0.0219 | 0.4847 | BMI |
| rs10830963 | 11 | MTNR1B | C | G | 0.724 | -0.095 | 0.007 | 5.00E-43 | 0.0353 | 0.019 | 0.0638 |  |
| rs10842994 | 12 | RN7SKP15 | T | C | 0.195 | -0.077 | 0.008 | 4.00E-20 | -0.0118 | 0.0219 | 0.5913 |  |
| rs10882101 | 10 | HHEX | T | C | 0.587 | 0.058 | 0.010 | 1.00E-08 | 0.0296 | 0.0155 | 0.0564 |  |
| rs10908278 | 17 | HNF1B | A | T | 0.519 | -0.077 | 0.006 | 6.00E-36 | 0.0042 | 0.0165 | 0.7997 |  |
| rs10938398 | 4 | NMU | A | G | 0.429 | 0.049 | 0.007 | 4.00E-12 | -0.0163 | 0.016 | 0.3098 | BMI |
| rs10954772 | 8 | PURG | T | C | 0.314 | 0.039 | 0.007 | 2.00E-09 | 0.011 | 0.0169 | 0.5128 | BMI |
| rs10974438 | 9 | GLIS3 | A | C | 0.643 | -0.049 | 0.006 | 2.00E-14 | -0.0283 | 0.0168 | 0.0926 |  |
| rs11063028 | 12 | CCND2-AS1 | T | C | 0.820 | -0.058 | 0.009 | 9.00E-11 | 0.037 | 0.0206 | 0.0733 |  |
| rs11257655 | 10 | RN7SL232P | T | C | 0.218 | 0.086 | 0.007 | 2.00E-32 | 0.0072 | 0.0187 | 0.7019 | BMI |
| rs1127215 | 1 | PTGFRN | T | C | 0.416 | -0.049 | 0.007 | 2.00E-13 | -0.012 | 0.0156 | 0.4400 |  |
| rs11496066 | 7 | FBXL13 | T | C | 0.818 | 0.077 | 0.013 | 1.00E-08 | 0.0187 | 0.0199 | 0.3478 |  |
| rs115505614 | 5 | GIN1 | T | C | 0.050 | 0.174 | 0.015 | 1.00E-30 | 0.0479 | 0.0424 | 0.2593 |  |
| rs11642430 | 16 | FAM57B | C | G | 0.601 | -0.039 | 0.007 | 2.00E-09 | -0.0075 | 0.0159 | 0.6378 | BMI |
| rs11680058 | 2 | AC010880.1 | A | G | 0.863 | 0.058 | 0.010 | 1.00E-08 | 0.0162 | 0.0258 | 0.5299 |  |
| rs11688682 | 2 | AC073257.1 | C | G | 0.272 | -0.049 | 0.008 | 4.00E-09 | -0.0189 | 0.022 | 0.3893 |  |
| rs11699802 | 20 | - | T | C | 0.464 | -0.039 | 0.006 | 2.00E-11 | 0.0164 | 0.0154 | 0.2889 |  |
| rs11708067 | 3 | ADCY5 | A | G | 0.772 | 0.086 | 0.007 | 5.00E-32 | -0.0146 | 0.0182 | 0.4229 | BMI |
| rs11709077 | 3 | PPARG | A | G | 0.124 | -0.131 | 0.010 | 2.00E-36 | 0.0153 | 0.0235 | 0.5153 | BMI |
| rs11759026 | 6 | RP11-394G3.2 | A | G | 0.768 | -0.068 | 0.008 | 2.00E-18 | 0.0191 | 0.0186 | 0.3031 |  |
| rs11842871 | 13 | HMGB1 | T | G | 0.266 | -0.039 | 0.007 | 1.00E-08 | 0.0173 | 0.0195 | 0.3752 | BMI |
| rs12001437 | 9 | RP11-537H15.3 | T | C | 0.628 | -0.039 | 0.006 | 3.00E-10 | 0.0356 | 0.0159 | 0.0251 |  |
| rs12140153 | 1 | PATJ | T | G | 0.095 | -0.068 | 0.012 | 1.00E-08 | 0.0615 | 0.0334 | 0.0651 | BMI |
| rs1260326 | NA | GCKR | T | C | 0.393 | -0.068 | 0.007 | 7.00E-25 | -5.00E-04 | 0.0157 | 0.9748 |  |
| rs12640250 | 4 | MTCO3P44 | A | C | 0.285 | -0.039 | 0.007 | 4.00E-08 | -0.0068 | 0.0174 | 0.6939 |  |
| rs12719778 | 8 | AF186192.1 | T | C | 0.538 | 0.039 | 0.007 | 5.00E-09 | 0.0014 | 0.0155 | 0.9287 |  |
| rs12811407 | 12 | FBRSL1 | A | G | 0.331 | 0.049 | 0.007 | 2.00E-12 | -0.0209 | 0.0172 | 0.2239 |  |
| rs12920022 | 16 | SPG7 | A | T | 0.158 | 0.049 | 0.008 | 3.00E-09 | 0.0212 | 0.0233 | 0.3629 |  |
| rs1296328 | 4 | RP11-775H9.2 | A | C | 0.446 | 0.039 | 0.007 | 4.00E-08 | -0.0437 | 0.0163 | 0.0074 |  |
| rs13041756 | 20 | RP5-984P4.1 | T | C | 0.893 | -0.058 | 0.010 | 1.00E-08 | -0.0167 | 0.024 | 0.4852 |  |
| rs13262861 | 8 | NKX6-3 | A | C | 0.171 | -0.068 | 0.010 | 4.00E-12 | -0.0294 | 0.0214 | 0.1701 |  |
| rs13426680 | 2 | CYTIP | A | G | 0.937 | 0.086 | 0.014 | 7.00E-10 | -0.0529 | 0.028 | 0.0591 |  |
| rs1359790 | 13 | RP11-470M1.2 | A | G | 0.280 | -0.086 | 0.007 | 2.00E-31 | 0.012 | 0.0171 | 0.4836 |  |
| rs1377807 | 17 | ZZEF1 | C | G | 0.312 | 0.049 | 0.007 | 4.00E-13 | 0.0341 | 0.0172 | 0.0466 |  |
| rs1412234 | 9 | LINGO2 | T | C | 0.677 | -0.039 | 0.006 | 2.00E-10 | -0.0017 | 0.0169 | 0.9185 | BMI |
| rs141521721 | 11 | PDE3B | A | C | 0.024 | 0.122 | 0.022 | 3.00E-08 | 0.0606 | 0.0556 | 0.2757 |  |
| rs1421085 | 16 | FTO | T | C | 0.585 | -0.122 | 0.006 | 3.00E-84 | -0.0322 | 0.0165 | 0.0507 | BMI |
| rs1426371 | 12 | WSCD2 | A | G | 0.261 | -0.049 | 0.007 | 8.00E-12 | -0.0283 | 0.0188 | 0.1314 |  |
| rs145678014 | 11 | QSER1 | T | G | 0.043 | -0.104 | 0.016 | 2.00E-10 | -0.0381 | 0.0472 | 0.4187 |  |
| rs145904381 | 1 | BNIPL | T | C | 0.987 | 0.174 | 0.031 | 3.00E-08 | 0.1066 | 0.0716 | 0.1367 |  |
| rs1493694 | 1 | NOTCH2 | T | C | 0.109 | 0.086 | 0.011 | 3.00E-16 | -0.0068 | 0.0248 | 0.784 |  |
| rs1531583 | 4 | PCGF3 | T | G | 0.046 | 0.122 | 0.016 | 4.00E-14 | -0.0435 | 0.0373 | 0.2434 |  |
| rs1561927 | 8 | LINC00824 | T | C | 0.731 | -0.039 | 0.007 | 2.00E-09 | -0.0063 | 0.0176 | 0.7184 |  |
| rs1562396 | 7 | RP11-36B6.1 | A | G | 0.681 | -0.058 | 0.007 | 1.00E-17 | 0.0406 | 0.0169 | 0.0163 |  |
| rs1580278 | 4 | RP11-119H12.3 | A | C | 0.527 | -0.039 | 0.006 | 2.00E-10 | 0.0059 | 0.0162 | 0.7166 |  |
| rs1708302 | 7 | JAZF1 | T | C | 0.488 | -0.095 | 0.006 | 1.00E-48 | 0.0309 | 0.0155 | 0.0463 |  |
| rs17122772 | 14 | SLC7A7 | C | G | 0.772 | -0.039 | 0.007 | 2.00E-08 | -0.0286 | 0.0205 | 0.1627 |  |
| rs17168486 | 7 | DGKB | T | C | 0.181 | 0.068 | 0.008 | 2.00E-17 | 0.0033 | 0.0193 | 0.8656 |  |
| rs17250977 | 5 | ANKH | A | G | 0.962 | -0.113 | 0.017 | 2.00E-11 | 0.0383 | 0.0728 | 0.5994 |  |
| rs17522122 | 14 | AKAP6 | T | G | 0.474 | 0.039 | 0.007 | 3.00E-09 | 0.0376 | 0.0156 | 0.0163 | BMI |
| rs17684074 | 18 | WDR7 | C | G | 0.260 | -0.039 | 0.007 | 3.00E-08 | 1.00E-04 | 0.0181 | 0.9940 |  |
| rs17772814 | 8 | CASC11 | A | G | 0.085 | -0.077 | 0.012 | 5.00E-10 | -0.01 | 0.0421 | 0.8121 |  |
| rs17791513 | 9 | CHCHD2P9 | A | G | 0.932 | 0.095 | 0.013 | 3.00E-14 | 0.0572 | 0.0291 | 0.0491 |  |
| rs1783541 | 11 | SCYL1 | T | C | 0.204 | 0.058 | 0.008 | 2.00E-14 | 0.0592 | 0.02 | 0.0031 | BMI |
| rs17836088 | 14 | NRXN3 | C | G | 0.217 | 0.058 | 0.008 | 7.00E-14 | 0.0329 | 0.0189 | 0.0811 | BMI |
| rs1796330 | 12 | TSPAN8 | C | G | 0.429 | -0.049 | 0.006 | 2.00E-14 | -0.0067 | 0.0153 | 0.6614 |  |
| rs1800961 | 20 | HNF4A | T | C | 0.035 | 0.166 | 0.017 | 2.00E-22 | 0.0433 | 0.0418 | 0.2999 |  |
| rs184509201 | 10 | TCF7L2 | C | G | 0.982 | 0.191 | 0.026 | 1.00E-13 | -0.06 | 0.0692 | 0.3863 |  |
| rs1903002 | 4 | FAM13A | C | G | 0.500 | -0.039 | 0.007 | 3.00E-08 | -0.0063 | 0.0158 | 0.6919 |  |
| rs2102278 | 4 | NMU | A | G | 0.681 | -0.039 | 0.007 | 4.00E-08 | 0.0147 | 0.0171 | 0.3882 |  |
| rs2197973 | 12 | USP44 | T | C | 0.538 | 0.039 | 0.007 | 4.00E-08 | -0.0081 | 0.0158 | 0.6105 |  |
| rs2237895 | 11 | KCNQ1 | A | C | 0.574 | -0.113 | 0.007 | 6.00E-52 | 0.0491 | 0.0167 | 0.0033 | BMI |
| rs2258238 | 12 | HMGA2 | A | T | 0.896 | -0.095 | 0.010 | 5.00E-21 | -0.0253 | 0.0246 | 0.3032 |  |
| rs2268078 | 20 | RALY | A | G | 0.657 | 0.039 | 0.006 | 2.00E-10 | 0.0297 | 0.0166 | 0.0730 |  |
| rs2272163 | 3 | ROBO2 | A | C | 0.382 | -0.039 | 0.007 | 1.00E-08 | -0.0184 | 0.0159 | 0.2477 |  |
| rs2283220 | 11 | KCNQ1 | A | G | 0.690 | 0.049 | 0.008 | 1.00E-09 | 0.0152 | 0.0184 | 0.4113 |  |
| rs2307111 | 5 | POC5 | T | C | 0.605 | 0.049 | 0.006 | 2.00E-16 | -0.003 | 0.0156 | 0.8465 | BMI |
| rs243024 | 2 | AC007381.3 | A | G | 0.460 | 0.058 | 0.006 | 3.00E-20 | 0.0039 | 0.0154 | 0.7987 |  |
| rs2767036 | 11 | - | A | C | 0.709 | -0.039 | 0.007 | 3.00E-08 | -0.0106 | 0.0164 | 0.5171 |  |
| rs2796441 | 9 | TLE1 | A | G | 0.408 | -0.068 | 0.007 | 4.00E-24 | -0.0211 | 0.0159 | 0.1845 |  |
| rs28505901 | 9 | GPSM1 | A | G | 0.248 | -0.086 | 0.008 | 7.00E-26 | 0.0031 | 0.0264 | 0.9060 |  |
| rs2972144 | 2 | NEU2 | A | G | 0.362 | -0.095 | 0.007 | 2.00E-46 | 0.0113 | 0.0164 | 0.4912 |  |
| rs3111316 | 19 | FARSA | A | G | 0.589 | 0.049 | 0.007 | 6.00E-13 | 0.0095 | 0.0157 | 0.5470 |  |
| rs329122 | 5 | JADE2 | A | G | 0.429 | 0.039 | 0.007 | 4.00E-09 | 0.0043 | 0.0153 | 0.7769 | BMI |
| rs340874 | 1 | PROX1 | T | C | 0.445 | -0.068 | 0.007 | 2.00E-22 | -0.0108 | 0.0155 | 0.4867 |  |
| rs34584161 | 13 | RNF6 | A | G | 0.760 | 0.049 | 0.008 | 2.00E-10 | 0.0081 | 0.0178 | 0.6491 |  |
| rs34715063 | 15 | RASGRP1 | T | C | 0.877 | -0.095 | 0.011 | 2.00E-19 | -0.0234 | 0.0282 | 0.4074 |  |
| rs348330 | 1 | ABCB10 | A | G | 0.640 | -0.049 | 0.006 | 3.00E-14 | 0.0097 | 0.0166 | 0.5606 |  |
| rs34965774 | 12 | KSR2 | A | G | 0.144 | 0.058 | 0.010 | 2.00E-09 | 0.0083 | 0.0221 | 0.7069 |  |
| rs35352848 | 3 | UBE2E2 | T | C | 0.788 | 0.068 | 0.008 | 1.00E-17 | -0.0074 | 0.0196 | 0.7067 |  |
| rs35895680 | 17 | RP11-501C14.5 | A | C | 0.322 | -0.058 | 0.007 | 3.00E-15 | -0.0293 | 0.0173 | 0.0913 | BMI |
| rs35999103 | 2 | AC062032.1 | T | C | 0.155 | 0.049 | 0.009 | 1.00E-08 | -0.017 | 0.0204 | 0.4028 |  |
| rs3751837 | 16 | CLUAP1 | T | C | 0.220 | 0.039 | 0.007 | 1.00E-08 | 0.0085 | 0.0189 | 0.6523 |  |
| rs3768321 | 1 | PABPC4 | T | G | 0.200 | 0.086 | 0.008 | 3.00E-26 | -0.0263 | 0.0212 | 0.2136 | BMI |
| rs3798519 | 6 | TFAP2B | A | C | 0.816 | -0.058 | 0.008 | 3.00E-12 | -0.0396 | 0.019 | 0.0367 | BMI |
| rs3802177 | 8 | SLC30A8 | A | G | 0.315 | -0.104 | 0.007 | 1.00E-55 | 0.0069 | 0.0169 | 0.6833 |  |
| rs3845281 | 5 | FAM105A | A | G | 0.096 | -0.077 | 0.011 | 2.00E-11 | 0.0202 | 0.0281 | 0.4727 |  |
| rs3887925 | 3 | ST6GAL1 | T | C | 0.547 | 0.068 | 0.007 | 3.00E-22 | -0.0109 | 0.0155 | 0.4827 |  |
| rs4238013 | 12 | CCND2-AS1 | T | C | 0.791 | -0.058 | 0.009 | 3.00E-11 | -0.02 | 0.0219 | 0.3603 |  |
| rs429358 | NA | APOE | T | C | 0.846 | 0.077 | 0.009 | 3.00E-18 | 0.0114 | 0.0231 | 0.6219 | BMI |
| rs4457053 | 5 | ZBED3-AS1 | A | G | 0.696 | -0.058 | 0.007 | 8.00E-18 | -0.023 | 0.0171 | 0.1786 |  |
| rs465002 | 5 | - | T | C | 0.742 | 0.104 | 0.008 | 6.00E-38 | 3.00E-04 | 0.018 | 0.9855 |  |
| rs4688760 | 3 | RBM6 | T | C | 0.684 | 0.039 | 0.006 | 4.00E-10 | 0.0163 | 0.0168 | 0.3321 | BMI |
| rs4709746 | 6 | RP1-230L10.1 | T | C | 0.132 | -0.058 | 0.010 | 6.00E-09 | 0.0278 | 0.0218 | 0.2030 |  |
| rs474513 | 6 | SLC22A3 | A | G | 0.517 | 0.039 | 0.006 | 8.00E-10 | -9.00E-04 | 0.0153 | 0.9511 |  |
| rs4804833 | 19 | MAP2K7 | A | G | 0.390 | 0.049 | 0.007 | 8.00E-13 | 0.0269 | 0.0159 | 0.0905 |  |
| rs4925109 | 17 | RAI1 | A | G | 0.316 | 0.049 | 0.007 | 3.00E-12 | -0.0169 | 0.0163 | 0.2987 |  |
| rs4929965 | 11 | TH | A | G | 0.383 | 0.068 | 0.006 | 4.00E-26 | 0.0652 | 0.0165 | 8.21E-05 |  |
| rs4932265 | 15 | C15orf38-AP3S2 | T | C | 0.267 | 0.068 | 0.007 | 4.00E-20 | -0.0162 | 0.0182 | 0.3732 |  |
| rs4946812 | 6 | BEND3 | A | G | 0.326 | -0.039 | 0.007 | 8.00E-09 | -0.0292 | 0.0212 | 0.1676 |  |
| rs4977213 | 8 | BOP1 | T | C | 0.625 | -0.049 | 0.007 | 9.00E-14 | -0.0139 | 0.0161 | 0.3872 |  |
| rs505922 | 9 | ABO | T | C | 0.668 | -0.049 | 0.007 | 4.00E-12 | -0.0345 | 0.0163 | 0.0342 |  |
| rs539515 | 1 | SEC16B | A | C | 0.802 | -0.049 | 0.008 | 2.00E-10 | -0.0318 | 0.0193 | 0.0996 | BMI |
| rs55653563 | 9 | snoU13 | A | C | 0.732 | 0.039 | 0.007 | 2.00E-09 | 0.0206 | 0.0181 | 0.2562 |  |
| rs56337234 | 4 | ABLIM2 | T | C | 0.497 | -0.058 | 0.007 | 9.00E-18 | 0.0235 | 0.0191 | 0.2203 |  |
| rs56348580 | 12 | HNF1A | C | G | 0.311 | -0.049 | 0.007 | 2.00E-13 | 0.0065 | 0.0171 | 0.7014 |  |
| rs58432198 | 1 | FAF1 | T | C | 0.119 | -0.068 | 0.011 | 2.00E-10 | -0.001 | 0.0263 | 0.9698 |  |
| rs58730668 | 4 | ACSL1 | T | C | 0.858 | 0.068 | 0.009 | 1.00E-13 | -7.00E-04 | 0.0229 | 0.9757 |  |
| rs601945 | 6 | TBC1D22B | A | G | 0.823 | -0.058 | 0.011 | 5.00E-08 | -0.0273 | 0.0281 | 0.3307 |  |
| rs60276348 | 17 | ERN1 | T | C | 0.140 | 0.049 | 0.009 | 3.00E-08 | 0.0189 | 0.0223 | 0.3968 |  |
| rs6070625 | 20 | GNAS-AS1 | C | G | 0.483 | -0.049 | 0.006 | 5.00E-14 | 0.0132 | 0.0154 | 0.3938 |  |
| rs61676547 | 17 | BPTF | C | G | 0.192 | 0.058 | 0.009 | 3.00E-11 | 0.0043 | 0.0197 | 0.8254 | BMI |
| rs62007683 | 14 | MARK3 | T | G | 0.347 | -0.039 | 0.007 | 3.00E-08 | -0.0094 | 0.0164 | 0.5668 | BMI |
| rs62080313 | 18 | RP11-687D19.1 | T | C | 0.877 | -0.058 | 0.010 | 1.00E-08 | -0.002 | 0.0232 | 0.9312 |  |
| rs62107261 | 2 | AC105393.2 | T | C | 0.954 | 0.113 | 0.016 | 4.00E-12 | -0.0252 | 0.0604 | 0.6766 | BMI |
| rs62271373 | 3 | SIAH2 | A | T | 0.055 | 0.086 | 0.014 | 1.00E-09 | -0.0157 | 0.0358 | 0.6620 |  |
| rs6458354 | 6 | RP11-344J7.4 | T | C | 0.711 | -0.049 | 0.007 | 2.00E-12 | 0.006 | 0.0173 | 0.7285 |  |
| rs6459733 | 7 | UBE3C | C | G | 0.327 | -0.058 | 0.007 | 2.00E-17 | -0.0257 | 0.0172 | 0.1342 |  |
| rs6518681 | 22 | ISX | A | G | 0.086 | -0.086 | 0.012 | 1.00E-12 | 0.0149 | 0.0295 | 0.6126 |  |
| rs6600191 | 16 | FAM234A | T | C | 0.825 | 0.058 | 0.008 | 9.00E-13 | -0.0428 | 0.019 | 0.0243 |  |
| rs67232546 | 11 | ETS1 | T | C | 0.207 | 0.058 | 0.009 | 1.00E-11 | -8.00E-04 | 0.0198 | 0.9666 |  |
| rs6780171 | 3 | IGF2BP2 | A | T | 0.314 | 0.131 | 0.008 | 9.00E-56 | 4.00E-04 | 0.017 | 0.9827 | BMI |
| rs6821438 | 4 | RP11-363G15.2 | A | G | 0.534 | 0.039 | 0.006 | 4.00E-11 | 0.0097 | 0.0155 | 0.5325 |  |
| rs6976111 | 7 | CTTNBP2 | A | C | 0.313 | 0.039 | 0.007 | 1.00E-08 | 0.008 | 0.0175 | 0.6467 | SMOKING |
| rs7022807 | 9 | HAUS6 | A | G | 0.599 | -0.039 | 0.006 | 3.00E-10 | 0.007 | 0.0157 | 0.6556 |  |
| rs702634 | 5 | ARL15 | A | G | 0.690 | 0.049 | 0.007 | 8.00E-14 | 0.0025 | 0.0174 | 0.8881 |  |
| rs703972 | 10 | ZMIZ1 | C | G | 0.467 | -0.068 | 0.006 | 2.00E-29 | -0.0125 | 0.0155 | 0.4178 |  |
| rs71372253 | 17 | RP11-848P1.9 | T | C | 0.936 | -0.077 | 0.014 | 4.00E-08 | 0.0119 | 0.0396 | 0.7640 |  |
| rs7178762 | 15 | USP3 | T | C | 0.541 | -0.039 | 0.006 | 5.00E-10 | -0.0031 | 0.0154 | 0.8385 |  |
| rs7222481 | 17 | GLP2R | C | G | 0.324 | 0.039 | 0.007 | 1.00E-08 | 0.0144 | 0.0171 | 0.3990 |  |
| rs7240767 | 18 | LAMA1 | T | C | 0.624 | -0.039 | 0.007 | 2.00E-08 | 0.0023 | 0.0161 | 0.8865 |  |
| rs72802342 | 16 | CTRB2 | A | C | 0.077 | -0.157 | 0.013 | 4.00E-32 | -0.021 | 0.0328 | 0.5222 |  |
| rs72926932 | 18 | TCF4 | A | C | 0.916 | -0.086 | 0.011 | 1.00E-14 | 0.0238 | 0.0306 | 0.4367 |  |
| rs738408 | 22 | PNPLA3 | T | C | 0.226 | 0.049 | 0.008 | 1.00E-10 | 0.0107 | 0.0177 | 0.5450 |  |
| rs7629630 | 3 | EGFEM1P | A | T | 0.857 | 0.049 | 0.009 | 3.00E-08 | 0.0125 | 0.0224 | 0.5774 |  |
| rs7669833 | 4 | FHDC1 | A | T | 0.296 | -0.058 | 0.008 | 1.00E-14 | -0.0049 | 0.0187 | 0.7920 |  |
| rs76895963 | NA | CCND2 | T | G | 0.980 | 0.482 | 0.027 | 1.00E-69 | 0.0288 | 0.0726 | 0.6916 |  |
| rs7719891 | 5 | RASA1 | A | G | 0.742 | -0.039 | 0.007 | 2.00E-08 | -0.0031 | 0.0184 | 0.8600 |  |
| rs77464186 | 11 | ARAP1 | A | C | 0.836 | 0.104 | 0.009 | 5.00E-33 | -0.0108 | 0.0224 | 0.6276 |  |
| rs7756992 | NA | CDKAL1 | A | G | 0.727 | -0.140 | 0.007 | 2.00E-88 | -0.0037 | 0.017 | 0.8267 | BMI |
| rs77864822 | 12 | RMST | A | G | 0.932 | 0.077 | 0.013 | 1.00E-08 | -0.0379 | 0.0334 | 0.2555 |  |
| rs7903146 | 10 | TCF7L2 | T | C | 0.295 | 0.315 | NA | 1.00E-200 | 0.0028 | 0.0172 | 0.8688 | BMI |
| rs7987740 | 13 | LINC00370 | T | C | 0.609 | 0.039 | 0.007 | 4.00E-08 | -0.0203 | 0.0157 | 0.1977 |  |
| rs8010382 | 14 | PPP4R3A | A | G | 0.579 | -0.039 | 0.007 | 7.00E-09 | 0.0068 | 0.0158 | 0.6655 |  |
| rs80147536 | 2 | THADA | A | T | 0.904 | 0.122 | 0.011 | 3.00E-29 | 0.0028 | 0.0275 | 0.9185 |  |
| rs8037894 | 15 | NPM1P47 | C | G | 0.434 | -0.049 | 0.007 | 3.00E-13 | 0.0171 | 0.0155 | 0.2679 |  |
| rs8107974 | 19 | SUGP1 | A | T | 0.923 | -0.095 | 0.012 | 3.00E-15 | -5.00E-04 | 0.0291 | 0.9874 |  |
| rs878521 | 7 | CAMK2B | A | G | 0.245 | 0.058 | 0.008 | 2.00E-13 | 0.0201 | 0.0181 | 0.2662 |  |
| rs9379084 | 6 | RREB1 | A | G | 0.113 | -0.104 | 0.011 | 3.00E-21 | 0.016 | 0.0269 | 0.5508 |  |
| rs9430095 | 1 | SRGAP2 | C | G | 0.494 | 0.039 | 0.007 | 2.00E-08 | -0.0038 | 0.016 | 0.8113 |  |
| rs9494624 | 6 | NHEG1 | A | G | 0.290 | 0.039 | 0.007 | 6.00E-09 | 0.0242 | 0.0175 | 0.1653 |  |
| rs9563615 | 13 | RNY4P29 | A | T | 0.710 | 0.049 | 0.007 | 6.00E-11 | -0.013 | 0.017 | 0.4437 | BMI |
| rs9860730 | 3 | ADAMTS9-AS2 | A | G | 0.704 | 0.058 | 0.007 | 5.00E-15 | 0.0047 | 0.0168 | 0.7774 |  |
| rs9873618 | 3 | SLC2A2 | A | G | 0.290 | -0.068 | 0.007 | 5.00E-21 | 0.0132 | 0.0168 | 0.4331 | BMI |
| rs9957145 | 18 | GRP | A | G | 0.171 | -0.049 | 0.008 | 8.00E-09 | -0.0178 | 0.0201 | 0.3771 |  |

Chr: chromosome; Eaf: effect allele frequency; SE: standard error; SNP: single-nucleotide polymorphism

* in DIAGRAM; † in GLIDE; # Coefficient: ln (odds ratio)

132 SNPs were used as instrumental variables for type 2 diabetes.

**Supplementary Table 4.** Summary statistics for Mendelian randomization analysis of potential causal effect of type 2 diabetes (FinnGen) on periodontitis (GLADE)

| SNP | Chr | Nearest genes/contig | Effect allele | Other allele | Eaf† | Exposure: type 2 diabetes | | | Outcome: periodontitis  (21,982 cases vs. 41,944 controls) | | | Potential confounders |
| --- | --- | --- | --- | --- | --- | --- | --- | --- | --- | --- | --- | --- |
|  |  |  |  |  |  | Coefficient^#^ | SE | P value | Coefficient^#^ | SE | P value |  |
| rs10184004 | 2 | COBLL1 | T | C | 0.3576 | -0.0668 | 0.0108 | 7.31E-10 | 0.0274 | 0.0158 | 0.0821 |  |
| rs10408179 | 19 | RN7SL836P | C | T | 0.4465 | -0.0593 | 0.0105 | 1.39E-08 | -0.024 | 0.0167 | 0.1506 |  |
| rs1046317 | 4 | WFS1 | C | T | 0.6107 | 0.081 | 0.0107 | 3.09E-14 | -9.00E-04 | 0.0169 | 0.9553 |  |
| rs10830963 | 11 | MTNR1B | G | C | 0.3567 | 0.1183 | 0.0108 | 8.92E-28 | -0.0353 | 0.019 | 0.0638 |  |
| rs10882099 | 10 | HHEX | C | T | 0.4769 | -0.0818 | 0.0104 | 3.21E-15 | -0.0277 | 0.0156 | 0.0757 |  |
| rs10938397 | 4 | NMU | G | A | 0.4736 | 0.0682 | 0.0104 | 5.20E-11 | -0.0175 | 0.016 | 0.2739 | BMI |
| rs10965247 | 9 | CDKN2B-AS1 | G | A | 0.1518 | -0.1276 | 0.0145 | 1.64E-18 | -0.0122 | 0.0215 | 0.5700 | BMI |
| rs10974438 | 9 | GLIS3 | C | A | 0.3757 | 0.0584 | 0.0107 | 4.90E-08 | 0.0283 | 0.0168 | 0.0926 |  |
| rs112108223 | 12 | RP11-320N7.1 | A | G | 0.02236 | -0.3623 | 0.0365 | 2.89E-23 | -0.0342 | 0.1132 | 0.7625 |  |
| rs11257659 | 10 | - | T | C | 0.2648 | 0.0827 | 0.0118 | 2.64E-12 | 0.0055 | 0.0187 | 0.7672 |  |
| rs11263763 | 17 | HNF1B | G | A | 0.3543 | 0.0684 | 0.0109 | 3.06E-10 | -0.0064 | 0.0163 | 0.6946 |  |
| rs112694524 | 2 | ZFP36L2 | A | G | 0.03344 | -0.1734 | 0.0294 | 3.46E-09 | -0.0032 | 0.0321 | 0.9195 |  |
| rs115018313 | 6 | HLA-DQA2 | C | T | 0.05032 | 0.3246 | 0.024 | 1.01E-41 | 0.034 | 0.1082 | 0.7535 |  |
| rs11558471 | 8 | SLC30A8 | G | A | 0.3784 | -0.0753 | 0.0107 | 1.90E-12 | 0.0097 | 0.0168 | 0.5627 |  |
| rs11665052 | 18 | RP11-795H16.2 | G | A | 0.1947 | 0.0775 | 0.0131 | 3.01E-09 | 0.0138 | 0.0181 | 0.4439 | Alcohol/BMI |
| rs11712037 | 3 | PPARG | G | C | 0.1706 | -0.1087 | 0.0138 | 3.60E-15 | 0.0144 | 0.0234 | 0.5393 |  |
| rs11719201 | 3 | - | T | C | 0.1762 | -0.0886 | 0.0137 | 8.85E-11 | 0.0141 | 0.0181 | 0.4359 |  |
| rs11833342 | 12 | RPSAP52 | G | T | 0.05631 | 0.1525 | 0.0226 | 1.44E-11 | 0.0177 | 0.026 | 0.4962 |  |
| rs12449219 | 16 | RP11-538I12.2 | G | C | 0.05841 | 0.1342 | 0.0223 | 1.62E-09 | -0.0215 | 0.0237 | 0.3631 |  |
| rs139640586 | 2 | LINC01122 | C | A | 0.4088 | -0.0578 | 0.0106 | 4.24E-08 | -0.0098 | 0.0162 | 0.5435 |  |
| rs1397566 | 12 | TSPAN8 | G | A | 0.4275 | -0.0596 | 0.0105 | 1.25E-08 | 0.0014 | 0.0156 | 0.9302 |  |
| rs144155527 | 10 | TCF7L2 | T | C | 0.02356 | -0.2116 | 0.0345 | 8.87E-10 | 0.0591 | 0.069 | 0.3914 |  |
| rs1515110 | 2 | NEU2 | T | G | 0.6174 | 0.0752 | 0.0107 | 1.67E-12 | -0.0059 | 0.0164 | 0.7174 | BMI |
| rs17039732 | 2 | AC007381.3 | A | T | 0.0476 | 0.1481 | 0.0243 | 1.16E-09 | 0.0231 | 0.0326 | 0.4789 |  |
| rs1815311 | 6 | RREB1 | G | A | 0.4053 | 0.062 | 0.0106 | 5.21E-09 | -0.0039 | 0.0177 | 0.8267 |  |
| rs182788819 | 10 | RP11-242G20.1 | T | C | 0.03873 | 0.1488 | 0.0269 | 3.16E-08 | -0.0602 | 0.1101 | 0.5844 |  |
| rs2237897 | 11 | KCNQ1 | T | C | 0.08139 | -0.1782 | 0.0192 | 2.08E-20 | 0.0034 | 0.0311 | 0.9121 | BMI |
| rs2303700 | 19 | MAP2K7 | C | T | 0.6741 | -0.0649 | 0.0111 | 5.08E-09 | -0.0356 | 0.0185 | 0.0539 |  |
| rs28642213 | 9 | GPSM1 | G | A | 0.6974 | 0.1005 | 0.0113 | 6.25E-19 | -0.0067 | 0.0227 | 0.7665 |  |
| rs28752873 | 6 | - | C | T | 0.5064 | 0.0687 | 0.011 | 5.00E-10 | 0.0187 | 0.0224 | 0.4037 |  |
| rs3110641 | 17 | HNF1B | A | G | 0.2133 | -0.0715 | 0.0128 | 2.51E-08 | -0.0239 | 0.022 | 0.2769 |  |
| rs3887925 | 3 | ST6GAL1 | T | C | 0.463 | 0.0591 | 0.0104 | 1.40E-08 | -0.0109 | 0.0155 | 0.4827 |  |
| rs429358 | 19 | APOE | C | T | 0.1825 | -0.0821 | 0.0136 | 1.63E-09 | -0.0114 | 0.0231 | 0.6219 | BMI |
| rs45551238 | 20 | ATP5E | T | C | 0.04991 | -0.2345 | 0.0243 | 5.50E-22 | 0.1307 | 0.0959 | 0.1728 |  |
| rs4931017 | 12 | FGD4 | G | A | 0.6926 | 0.0625 | 0.0113 | 3.07E-08 | -0.0118 | 0.0189 | 0.5305 |  |
| rs498475 | 7 | JAZF1-AS1 | A | G | 0.6448 | -0.0625 | 0.0108 | 8.27E-09 | 0.0057 | 0.0158 | 0.7200 |  |
| rs5215 | 11 | KCNJ11 | T | C | 0.5284 | -0.0586 | 0.0104 | 1.62E-08 | -0.0068 | 0.0159 | 0.6700 | BMI |
| rs55993634 | 16 | CTRB2 | G | C | 0.08717 | -0.1449 | 0.0185 | 5.60E-15 | -0.0094 | 0.0275 | 0.7332 |  |
| rs56348580 | 12 | HNF1A | C | G | 0.2827 | -0.0786 | 0.0116 | 1.25E-11 | 0.0065 | 0.0171 | 0.7014 |  |
| rs57307671 | 7 | ORAI2 | T | A | 0.1835 | 0.0968 | 0.0134 | 6.04E-13 | -0.0069 | 0.0261 | 0.7906 |  |
| rs58102377 | 15 | RP11-307C19.2 | G | A | 0.4094 | -0.06 | 0.0106 | 1.41E-08 | -0.0096 | 0.0162 | 0.5553 | BMI |
| rs6073386 | 20 | GDAP1L1 | G | A | 0.03667 | 0.179 | 0.0273 | 5.93E-11 | 0.0307 | 0.0543 | 0.5718 |  |
| rs62137406 | 2 | THADA | T | C | 0.04896 | 0.1422 | 0.0239 | 2.57E-09 | -0.0564 | 0.085 | 0.5072 |  |
| rs62492368 | 7 | AOC1 | A | G | 0.3402 | 0.074 | 0.011 | 1.52E-11 | -0.0093 | 0.0166 | 0.5770 |  |
| rs6550758 | 3 | UBE2E2 | C | A | 0.8216 | -0.0826 | 0.0135 | 1.05E-09 | 0.0246 | 0.0183 | 0.1786 |  |
| rs6780171 | 3 | IGF2BP2 | A | T | 0.3066 | 0.0929 | 0.0112 | 1.22E-16 | 4.00E-04 | 0.017 | 0.9827 | BMI |
| rs6786846 | 3 | EIF5A2 | A | G | 0.681 | 0.0654 | 0.0111 | 4.18E-09 | -0.0112 | 0.0159 | 0.4797 | BMI |
| rs7018475 | 9 | CDKN2B-AS1 | G | T | 0.279 | 0.1126 | 0.0116 | 2.53E-22 | 0.0095 | 0.0238 | 0.6889 |  |
| rs73541184 | 11 | ARAP1 | A | G | 0.3083 | -0.0857 | 0.0113 | 2.75E-14 | 0.0119 | 0.0176 | 0.4976 |  |
| rs74862545 | 12 | CCND2-AS1 | T | C | 0.0195 | -0.2531 | 0.0388 | 6.88E-11 | -6.00E-04 | 0.0629 | 0.9922 |  |
| rs76177300 | 5 | PAM | A | G | 0.05784 | 0.1409 | 0.0222 | 2.32E-10 | 0.0476 | 0.0432 | 0.2708 |  |
| rs76895963 | 12 | CCND2 | G | T | 0.03116 | -0.4842 | 0.0325 | 2.93E-50 | -0.0288 | 0.0726 | 0.6916 |  |
| rs78470967 | 12 | FGF6 | A | T | 0.03944 | -0.2295 | 0.0273 | 4.33E-17 | -0.0356 | 0.046 | 0.4385 |  |
| rs7903146 | 10 | TCF7L2 | T | C | 0.1996 | 0.2969 | 0.0132 | 1.01E-112 | 0.0028 | 0.0172 | 0.8688 | BMI |
| rs7925578 | 11 | - | T | G | 0.3213 | 0.0736 | 0.0112 | 5.02E-11 | -0.0061 | 0.0174 | 0.7235 |  |
| rs7998259 | 13 | RP11-470M1.2 | A | G | 0.3894 | -0.0727 | 0.0107 | 1.07E-11 | 0.0207 | 0.0176 | 0.2394 |  |
| rs8100204 | 19 | SUGP1 | A | G | 0.1598 | 0.0956 | 0.0143 | 2.11E-11 | 0.0211 | 0.0245 | 0.3888 |  |
| rs8353 | 22 | KLHL22 | T | G | 0.3009 | -0.0686 | 0.0113 | 1.34E-09 | 0.0058 | 0.0183 | 0.7519 |  |
| rs878521 | 7 | CAMK2B | A | G | 0.2071 | 0.0784 | 0.0128 | 8.94E-10 | 0.0201 | 0.0181 | 0.2662 |  |
| rs9348441 | 6 | CDKAL1 | A | T | 0.3289 | 0.123 | 0.011 | 4.91E-29 | 0.0053 | 0.0175 | 0.7615 | BMI |
| rs9403134 | 6 | AL356739.1 | T | A | 0.1913 | -0.0774 | 0.0132 | 4.89E-09 | -0.017 | 0.0189 | 0.3668 |  |
| rs9933509 | 16 | FTO | C | T | 0.4129 | 0.1161 | 0.0105 | 2.62E-28 | 0.0313 | 0.0158 | 0.0480 | Alcohol/BMI |

Chr: chromosome; Eaf: effect allele frequency; SE: standard error; SNP: single-nucleotide polymorphism
* in FinnGen; † in GLIDE; # Coefficient: ln (odds ratio)

49 SNPs were used as instrumental variables for type 2 diabetes.

**Supplementary Table 5.** MR-PRESSO global test

| MR-PRESSO global test | RSSobs | P-value |
| --- | --- | --- |
| Using 5 instrumental SNPs for periodontitis (the GWAS of type 2 diabetes were obtained from DIAGRAM) | 5.847 | 0.560 |
| Using 5 instrumental SNPs for periodontitis (the GWAS of type 2 diabetes were obtained from FinnGen) | 39.924 | 0.837 |
| Using 132 instrumental SNPs from DIAGRAM for type 2 diabetes (the GWAS of periodontitis were obtained from GLIDE) | 152.373 | 0.127 |
| Using 49 instrumental SNPs from FinnGen for type 2 diabetes (the GWAS of periodontitis were obtained from GLIDE) | 39.924 | 0.837 |

GWAS, genome-wide association studies; DIAGRAM, the DIAbetes Genetics Replication And Meta-analysis; GLIDE, the Gene-Lifestyle Interactions and Dental Endpoints Consortium; RSSobs, observed residual sum of squares; SNP: single-nucleotide polymorphism

**Supplementary Table 6.** Post-hoc power calculations for Mendelian randomization analyses performed at varying causal effect sizes

|  |  |  | **Power (%)** | | | | |
| --- | --- | --- | --- | --- | --- | --- | --- |
|  | **N** | **K** | **Null causal effect** | **OR=1.05** | **OR=1.1** | **OR=1.2** | **OR=1.3** |
| Periodontitis on type 2 diabetes (the GWAS of type 2 diabetes were obtained from DIAGRAM) | 898130 | 0.08 | 5 | 99 | 100 | 100 | 100 |
| Chronic periodontitis (the GWAS of type 2 diabetes were obtained from FinnGen) | 215654 | 0.12 | 5 | 37 | 90 | 100 | 100 |
| Type 2 diabetes from DIAGRAM on periodontitis (the GWAS of chronic periodontitis were obtained from GLIDE) | 34615 | 0.35 | 5 | 67 | 100 | 100 | 100 |
| Type 2 diabetes from FinnGen on periodontitis (the GWAS of chronic were obtained from GLIDE) | 34615 | 0.35 | 5 | 79 | 100 | 100 | 100 |

N, sample size; K, proportion of cases in the study; OR, odds ratio; Type-1 error rate of 0.05; R^2^ for variance explained in the exposure

Post-hoc power calculations were performed using <https://shiny.cnsgenomics.com/mRnd>. We calculated power, using a Type-1 error rate of 0.05, based on achieved sample size in the outcome dataset and used an R^2^ for the variance explained in the exposure. We varied the “true” odds ratio of the outcome per standard deviation increase in the exposure from 1.0 (null causal effect), 1.05, 1.10, 1.20 and 1.30.


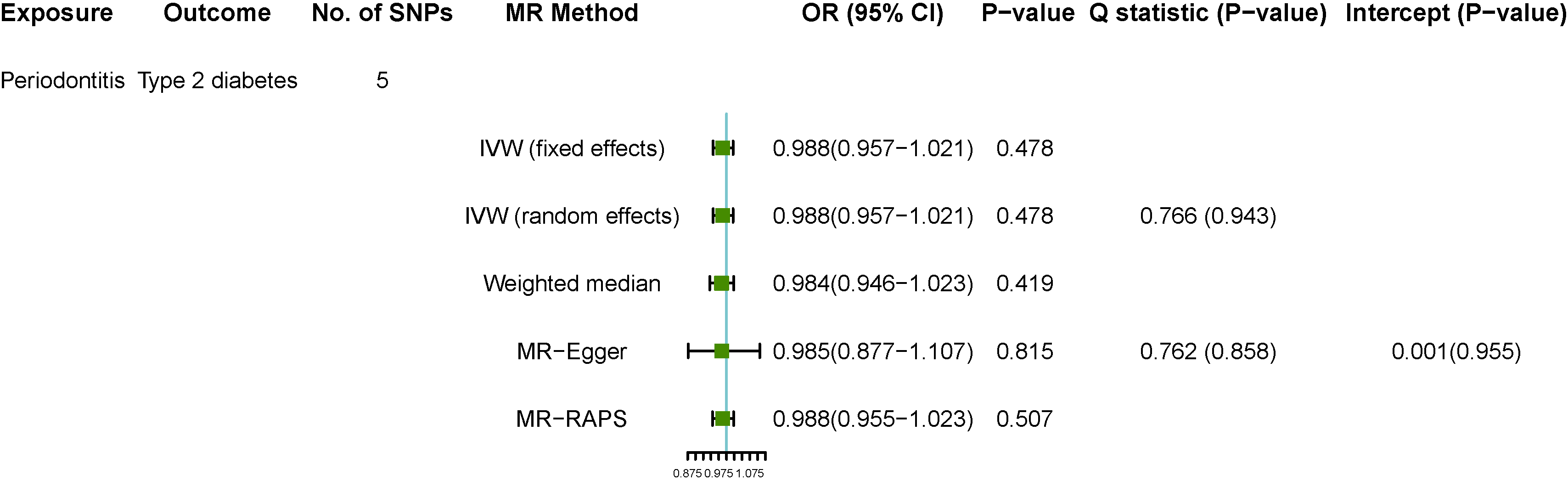


**Supplementary Figure 1.** Estimated causal effect of periodontitis on type 2 diabetes using different MR approaches. Using 5 instrumental SNPs for periodontitis (the summary statistics were obtained from FinnGen). Odds ratio (OR) represents the risk of type 2 diabetes per genetically determined 1-unit increase in ln (OR) of periodontitis. SNP: single-nucleotide polymorphism; MR: Mendelian randomization; CI: confidence interval of the odds ratio; IVW: inverse-variance weighted; RAPS: robust adjusted profile score.


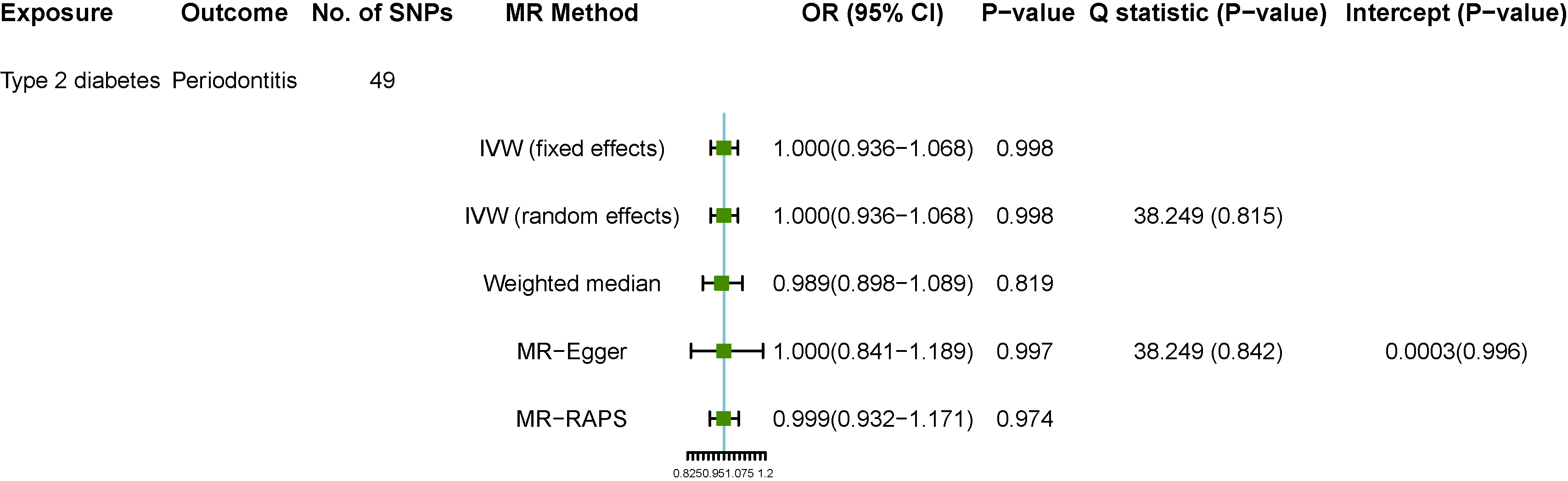


**Supplementary Figure 2.** Estimated causal effect of type 2 diabetes on periodontitis. Using 49 instrumental SNPs from FinnGen for type 2 diabetes. Odds ratio (OR) represents the risk of periodontitis per genetically determined 1-unit increase in ln (OR) of type 2 diabetes. SNP: single-nucleotide polymorphism; MR: Mendelian randomization; CI: confidence interval; IVW: inverse-variance weighted; RAPS: robust adjusted profile score.


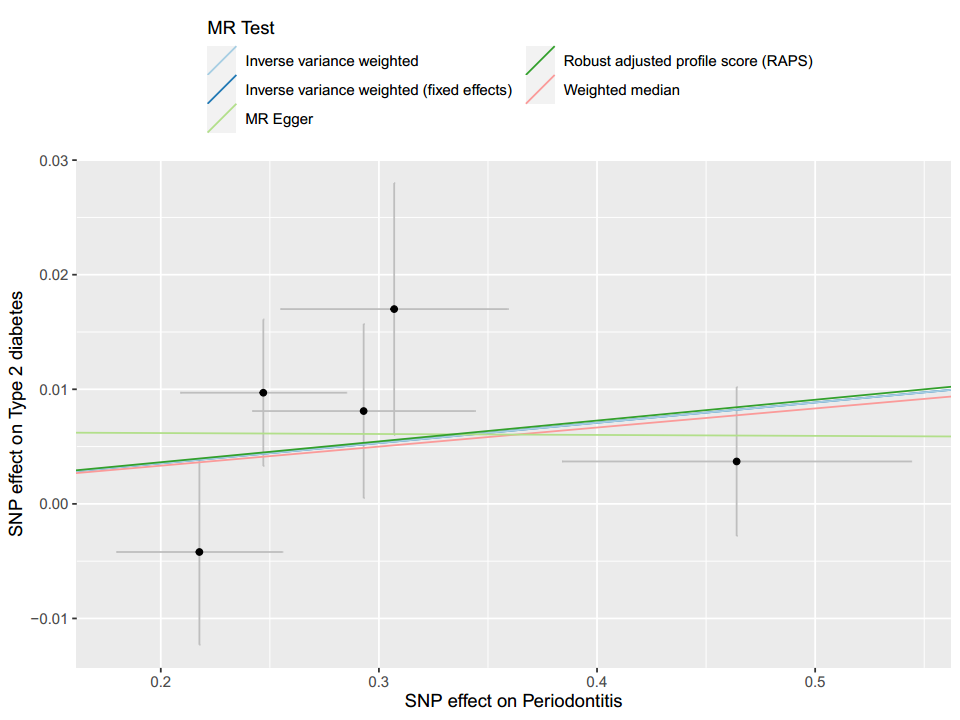


**Supplementary Figure 3.** Scatterplots comparing the strength of the SNP-exposure (periodontitis) and SNP-type 2 diabetes (DIAGRAM) associations. The lines indicate the estimated effect sizes by four Mendelian randomization methods (inverse‐variance weighted (IVW), Robust adjusted profile score(PAPS), MR Egger, and weighted median).


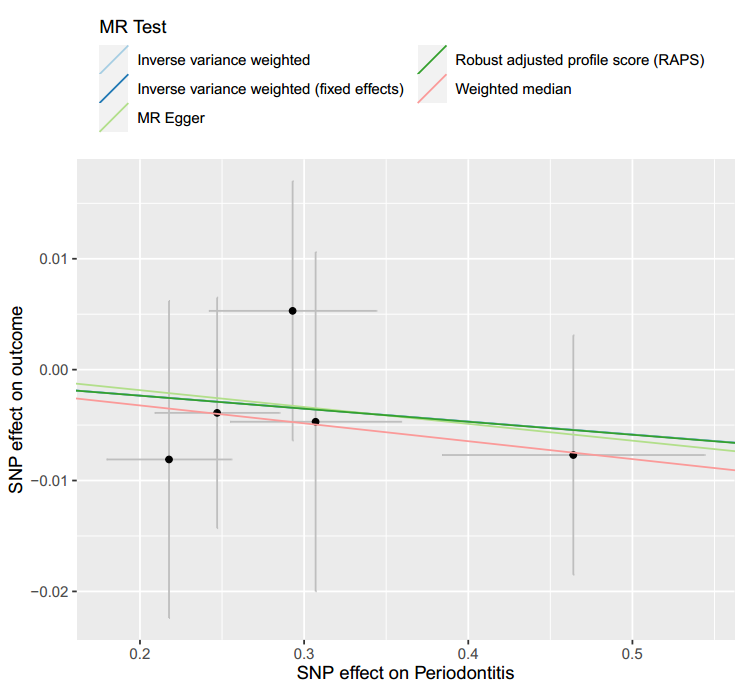


**Supplementary Figure 4.** Scatterplots comparing the strength of the SNP-exposure (periodontitis) and SNP-type 2 diabetes (FinnGen) associations. The lines indicate the estimated effect sizes by four Mendelian randomization methods (inverse‐variance weighted (IVW), Robust adjusted profile score (PAPS), MR Egger, and weighted median).


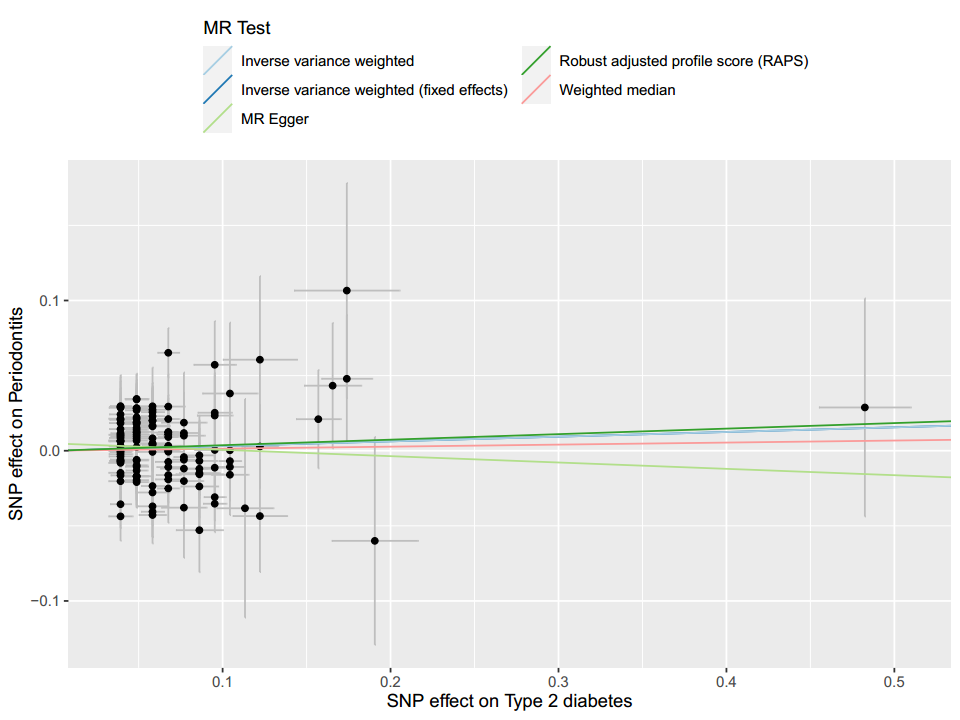


**Supplementary Figure 5.** Scatterplots comparing the strength of the SNP-exposure (type 2 diabetes in DIAGRAM) and SNP-periodontitis associations. The lines indicate the estimated effect sizes by four Mendelian randomization methods (inverse‐variance weighted (IVW), Robust adjusted profile score (PAPS), MR Egger, and weighted median).


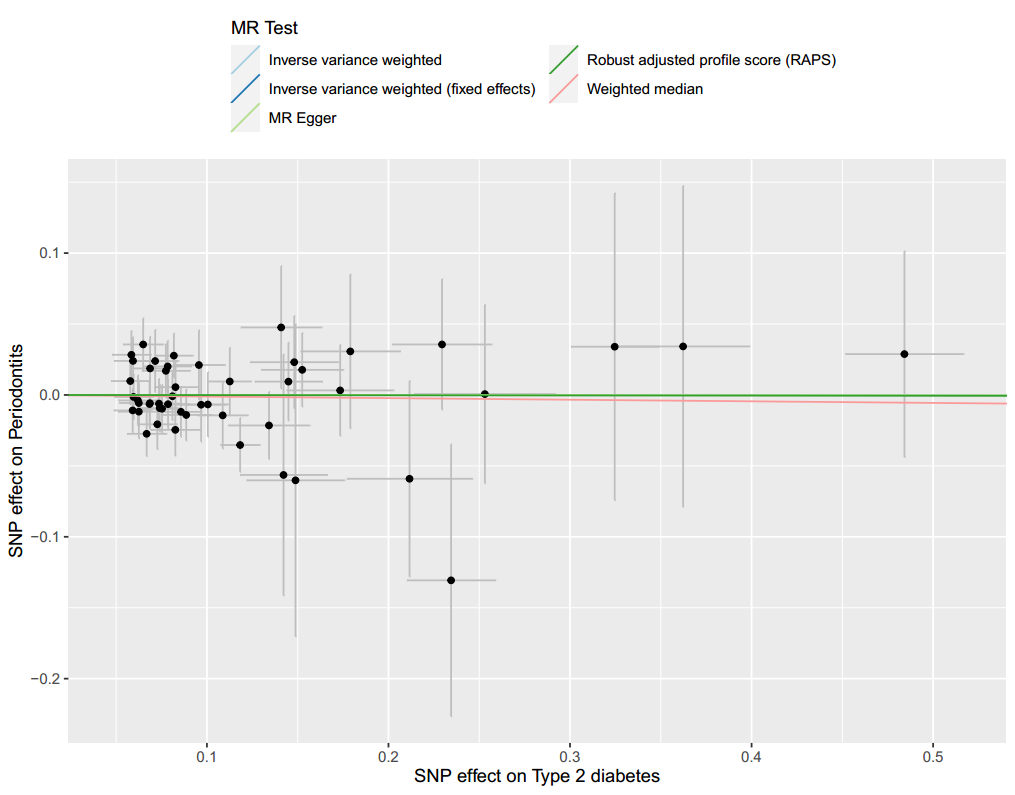


**Supplementary F****igure 6.** Scatterplots comparing the strength of the SNP-exposure (type 2 diabetes in FinnGen) and SNP-periodontitis associations. The lines indicate the estimated effect sizes by four Mendelian randomization methods (inverse‐variance weighted (IVW), Robust adjusted profile score (PAPS), MR Egger, and weighted median).


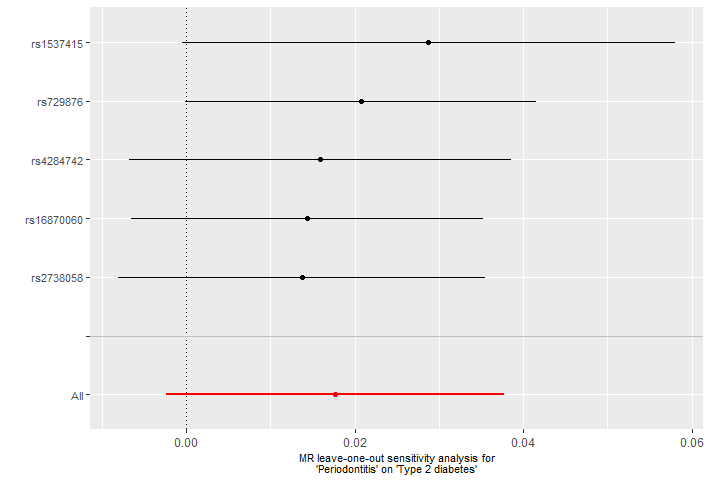


**Supplementary Figure 7**. Leave-one-out meta-analysis. The leave-one-out plot visualized how the causal estimates (point with horizontal line) for the effect of periodontitis on type 2 diabetes (DIAGRAM) were influenced by the removal of single variant.


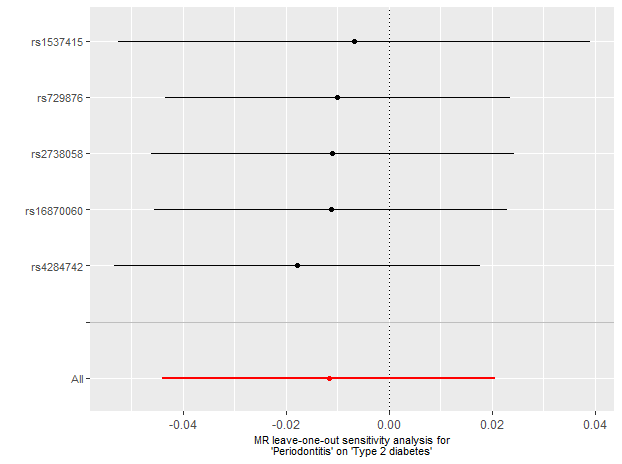


**Supplementary Figure 8**. Leave-one-out meta-analysis. The leave-one-out plot visualized how the causal estimates (point with horizontal line) for the effect of periodontitis on type 2 diabetes (FinnGen) were influenced by the removal of single variant.


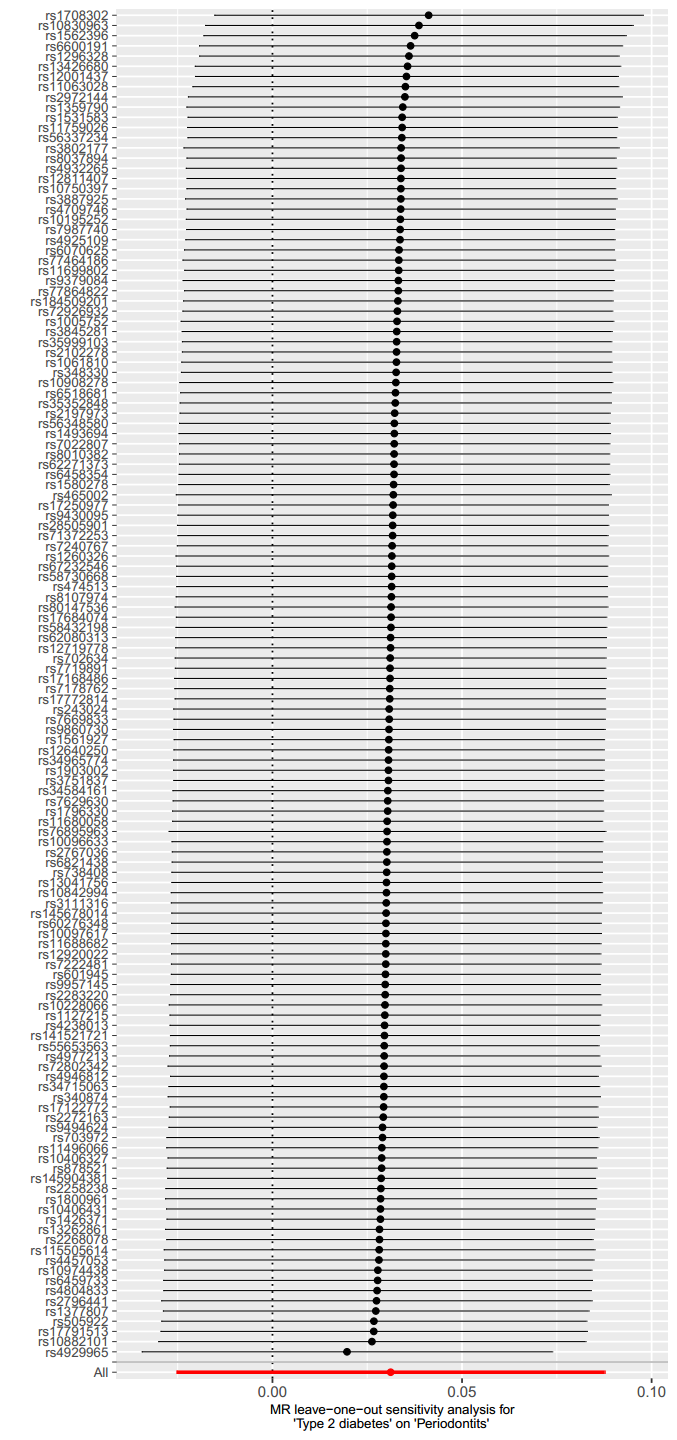


**Supplementary Figure 9**. Leave-one-out meta-analysis. The leave-one-out plot visualized how the causal estimates (point with horizontal line) for the effect of type 2 diabetes (DIAGRAM) on periodontitis were influenced by the removal of single variant.


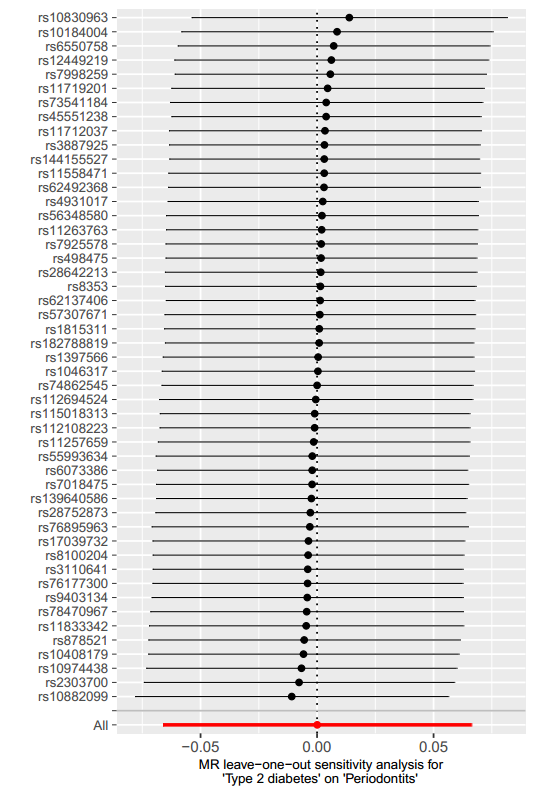


**Supplementary Figure 10**. Leave-one-out meta-analysis. The leave-one-out plot visualized how the causal estimates (point with horizontal line) for the effect of type 2 diabetes (FinnGen) on periodontitis were influenced by the removal of single variant.
